# Ethanolic extract of *Descurainia sophia* seeds sensitizes A549 human lung cancer cells to TRAIL cytotoxicity by upregulating death receptors

**DOI:** 10.1186/s12906-016-1094-0

**Published:** 2016-04-02

**Authors:** Jong-Shik Park, Chae Jun Lim, Ok-Sun Bang, No Soo Kim

**Affiliations:** KM-Convergence Research Division, Korea Institute of Oriental Medicine, 1672 Yuseong-daero, Yuseong-gu, Daejeon, 34054 Republic of Korea; Department of Korean Medicine, Life Science and Technology, Korea University of Science and Technology, Daejeon, Republic of Korea

**Keywords:** *Descurainia sophia*, TRAIL, Death receptor, CHOP, Sensitization

## Abstract

**Background:**

Our previous genome-wide gene expression analysis revealed that tumor necrosis factor-related apoptosis-inducing ligand (TRAIL) death receptors 4 (DR4) and 5 (DR5) are markedly upregulated by the ethanolic extract of *D. sohia* seeds (EEDS) in A549 TRAIL-refractory cancer cells. In the present study, we investigated whether the EEDS-mediated upregulation of TRAIL death receptors was associated with increased TRAIL-mediated toxicity in A549 cells in vitro.

**Methods:**

Cell proliferation and viability were determined by an automatic cell counter. Gene silencing was performed by introducing small interfering RNA into cells. Expression changes of cellular proteins were determined by western blot analysis. Apoptotic cell death was monitored by western blot analysis. Analysis of variance followed by the *post-hoc* Dunnett’s test was used to compare the data.

**Results:**

EEDS treatment increased both mRNA and protein levels of DR4 and DR5 in the TRAIL refractory A549 cells. Co-treatment of A549 cells with sub-lethal dose of EEDS and recombinant TRAIL increased the apoptotic cell death. Upregulation of DR5 by EEDS was mediated by an endoplasmic reticulum stress-induced transcription factor, CCAAT/enhancer-binding protein homologous protein (CHOP), and knockdown of CHOP expression inhibited EEDS-induced DR5 upregulation and abolished the EEDS-associated increase in TRAIL toxicity in A549 cells.

**Conclusions:**

EEDS can sensitize A549 cells to TRAIL cytotoxicity by upregulation of TRAIL death receptors. Our findings suggested that EEDS is a good initial herbal source for the development of an anticancer supplement for anticancer therapeutics associated with TRAIL.

**Electronic supplementary material:**

The online version of this article (doi:10.1186/s12906-016-1094-0) contains supplementary material, which is available to authorized users.

## Background

Cancer is the leading cause of death, and the prevalence of cancer continues to rise despite advances in earlier diagnosis, clinical intervention, and increased public awareness of the risk factors for cancer [1]. The mutation of regulatory genes that are involved in maintaining the equilibrium between cell proliferation and cell death creates cancer cells that undergo rapid, uncontrolled cell division and evade cell death [2]. Current standard cancer therapies involve surgery to remove the major tumor masses and subsequent radiotherapy and/or chemotherapy to kill potential remaining cancer cells and to prevent cancer recurrence. A drawback of these conventional radio-chemotherapies is their lack of specificity: they cannot discriminate cancer cells from normal cells, resulting in the substantial death of normal cells, which causes adverse side effects, such as gastro-intestinal complications, appetite loss, bone marrow/hematological complications, fatigue, weight loss, and pain [3]. The side effects may affect the quality of life of cancer patients, and moreover, they complicate the therapies and thereby affect the prognosis and survival of cancer patients [3]. Therefore, the ideal anticancer therapeutics should exclusively target cancer cells while sparing normal cells [4].

In the mid-1990s, tumor necrosis factor (TNF)-related apoptosis-inducing ligand (TRAIL), a new member of the TNF superfamily, was discovered [5, 6]. As a cytotoxic cytokine, TRAIL selectively induces apoptosis in tumor cells through homotrimeric binding to the membrane-bound death receptor 4 (DR4, also known as TRAIL-receptor 1 or TNF receptor superfamily member 10A) and death receptor 5 (DR5, also known as TRAIL-receptor 2 or TNF receptor superfamily member 10B), the recruitment of Fas-associated death domain protein (FADD), the formation of the death-inducing signaling complex (DISC), and the subsequent activation of caspase-8 and effector caspase-3 (extrinsic pathway). Activated caspase-8 also triggers the intrinsic apoptotic pathway by cleaving BH3-interacting domain death agonist (Bid); truncated Bid translocates to the mitochondria and induces cytochrome C release from the mitochondria, thereby activating caspase-9 and caspase-3 [7–9]. TRAIL is an attractive anticancer therapeutic due to its ability to induce apoptosis in a broad spectrum of cancer cells while sparing most normal cells [10–12]. However, the clinical application of TRAIL therapy has been limited by its inherently short half-life in blood (3–5 min in rodents and 24–31 min in non-human primates), insufficient delivery to the targets, and the appearance of cancer cell populations with intrinsic or acquired resistance to TRAIL-mediated programmed cell death [13, 14]. Improvements in the plasma half-life of TRAIL and delivery efficiency of TRAIL have been attempted by manipulating its structure, such as by creating fusions with the immunoglobulin Fc domain [15] or by coupling or encapsulating TRAIL into liposomes [14, 16]. The development of an agonistic monoclonal antibody (mAb) or chimeric antigen receptors specific for TRAIL receptors are additional potential strategies for overcoming TRAIL instability [17].

The issue of cancer cells acquiring TRAIL resistance can be overcome by the co- or pre-administration of agents that sensitize refractory cancer cells to TRAIL cytotoxicity. They include a heat shock protein 90 inhibitor NVP-AUY922 that suppresses JAK2-STAT3-Mcl-1 signaling pathway [8], a pan-histone deacetylase inhibitor trichostatin A isolated from *Streptomyces hygroscopius* that downregulates cellular FADD-like interleukin-1β-converting enzyme inhibitory protein (c-FLIP) [13], a phytochemical triptolide (PG490) isolated from *Tripterygium wilfordii* that activates mitogen-activated protein kinase ERK2 [18], a phytochemical carnosic acid isolated from *Rosmarinus officinalis* that upregulates death receptor DR5 [19], and a mitochondrial respiration inhibitor rotenone that reciprocally regulates DR5 (up-) and c-FLIP (down-) [20]. Certain previous reports have demonstrated that crude extracts or purified active phytochemicals from medicinal herbs with pharmacological activity exert synergistic cytotoxicity against cancer cells when co-administered with recombinant TRAIL [9, 21–23]. From our previous gene expression profiling of TRAIL-refractory A549 human lung cancer cells, we found that DR4 and DR5 expression was enhanced by treatment with the ethanolic extract of *Descurainia sophia* seeds (EEDS) [24]. In this study, we determined whether the EEDS-mediated upregulation of DR4 and DR5 translated to sensitization of A549 cells to TRAIL cytotoxicity. Our data suggested that CCAAT/enhancer-binding protein homologous protein (CHOP), an endoplasmic reticulum (ER) stress-induced transcription factor, was a critical regulator of the EEDS-mediated upregulation of TRAIL death receptors.

## Methods

### Plant materials and EEDS preparation

The dried seeds of *D. sophia* were obtained from Kwangmyungdang Medicinal Herbs Co. (Ulsan, Republic of Korea) and identified by Dr. Go Ya Choi, K-Herb Research Center, Korea Institute of Oriental Medicine, Daejeon, Republic of Korea. A voucher specimen (KIOM-CRC-5) was deposited at KM-Convergence Research Division, Korea Institute of Oriental Medicine. EEDS was prepared as described in our previous report [24]. In brief, the dried seeds of *D. sophia* (9 kg) were ground in an electric grinder and were subjected to solvent extraction with 80 % (v/v) of ethanol (40 L). The extraction was performed three times at room temperature. The extracts were filtered through a Whatman filter paper (No. 2, Whatman International, Maidstone, England) and were concentrated using a rotary evaporator (EYELA, Tokyo Rikakikai, Tokyo, Japan) at 40 °C. The sticky solid lower extract (535.7 g) was collected and further dried in a WiseVen vacuum oven (WOW-70, Daihan Scientific, Seoul, Republic of Korea) at 40 °C for 24 h. The vacuum-dried powder of EEDS was homogenized using a mortar, dissolved in 100 % dimethyl sulfoxide (DMSO, Sigma-Aldrich, St. Louis, MO, USA) to final concentration of 20 mg/mL, and sterilized by passage through 0.22 μm syringe filters (Millipore, Billerica, MA, USA). The sterilized EEDS stock solution was aliquoted in small volumes and stored at -80 °C.

### Cell culture and reagents

The A549 and NCI-H460 human non-small cell lung carcinoma (NSCLC) cell lines were directly obtained from American Type Culture Collection (Rockville, VA, USA). Lung cancer is the leading cause of cancer deaths in the Republic of Korea (http://kostat.go.kr) and we have tried to discover novel anticancer agents targeting lung cancer, especially NSCLC. We chose A549 and NCI-H460 cell lines in the present study because they were previously known as TRAIL-refractory (A549) and sensitive (NCI-H460) NSCLC cells [25, 26]. Authentication of the cell lines was done using a short tandem repeat analysis by Korean Cell Line Bank (Seoul National University College of Medicine, Seoul, Republic of Korea). These cell lines were cultured in RPMI1640 basal medium supplemented with 10 % (v/v) fetal bovine serum, 100 U/mL penicillin, and 100 mg/mL streptomycin. The cells were grown at 37 °C in a humidified incubator containing 5 % CO_2_. Cell growth and viability were determined using an ADAM-MC automatic cell counter (NanoEnTek, Seoul, Republic of Korea) as previously described [27]. All the supplements and the basal media for cell culture as well as recombinant TRAIL were obtained from Invitrogen (Carlsbad, CA, USA). TRAIL was dissolved in sterile phosphate-buffered saline (PBS) containing 0.1 % (w/v) bovine serum albumin (BSA) as a carrier protein.

### Western blot analysis

Total protein was prepared using RIPA cell lysis buffer (Thermo Scientific, Rockford, IL, USA) containing 100 μM phenylmethylsulfonyl fluoride (Sigma-Aldrich) and an ethylenediaminetetraacetic acid-free protease inhibitor cocktail (Roche, Mannheim, Germany). The protein concentration was determined by the bicinchoninic acid assay kit (Thermo Scientific). Proteins were separated by sodium dodecyl sulfate-polyacrylamide gel electrophoresis and blotted to nitrocellulose membranes (Millipore). The membranes were blocked with 5 % (w/v) skim milk in 0.1 % (v/v) Tween 20-PBS for 1 h. The blots were probed with primary antibodies against specific proteins at 4 °C overnight, and the primary antibodies were then captured with secondary antibodies conjugated to horseradish peroxidase (HRP) for 1 h. The immunoreactive bands were visualized with ECL (GE Healthcare, Piscataway, NJ, USA). Rabbit polyclonal anti-DR5 (#3696), -poly ADP ribose polymerase (PARP) (#9542), -caspase-9 (#9502), mouse monoclonal anti-caspase-8 (#9746, clone 1C12), -CHOP (#2895, clone L63F7), and rabbit monoclonal anti-caspase-3 (#9665, clone 8G10) were obtained from Cell Signaling Technology (Danvers, MA, USA). Rabbit polyclonal anti-DR4 (Ab8414) and mouse monoclonal anti-β-actin (A1978, clone Ac-15) antibodies were obtained from Abcam and Sigma-Aldrich, respectively.

### Gene silencing of CHOP

A549 cells were plated in a six-well culture plate (10^5^ cells/well) and incubated for 24 h. The cells were transiently transfected with CHOP-specific small interfering RNA (siRNA; sc-35437, Santa Cruz Biotechnology, CA, USA) or control siRNA (sc-37007, Santa Cruz Biotechnology) using Lipofectamine 2000 (Invitrogen) according to the user’s guide. After 48 h, the medium was replaced with fresh medium containing 5 μg/mL EEDS. The cells were further incubated for 24 h, and total protein extracts were prepared for western blot analysis to determine the efficiency of the CHOP siRNA.

### Statistical analysis

The results are expressed as the mean ± standard deviation (SD) of triplicate experiments. Differences in the continuous variables were analyzed by analysis of variance followed by the post-hoc Dunnett’s test. Values of *P* < 0.05 were considered significant.

## Results

### TRAIL cytotoxicity was cell type-dependent

**Fig. 1:**
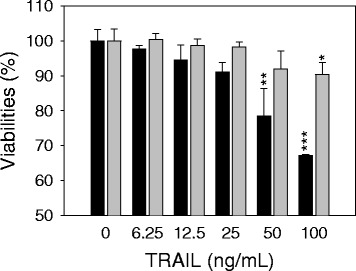
Differential sensitivities of lung cancer cells to TRAIL cytotoxicity. NCI-H460 (black bars) and A549 (gray bars) cells were exposed to increasing concentrations of TRAIL (0-20 μg/mL) for 48 h, and their viability was determined based on membrane integrity. The cell viability is presented relative to vehicle treatment (PBS containing 0.1 % BSA). The data are presented as the mean ± SD of triplicate experiments. *P <0.05, **P <0.01, ***P <0.01 vs. vehicle control

First, we tested the cytotoxicity of recombinant TRAIL in 2 different human NSCLC cell lines, A549 and NCI-H460. The cells were exposed to increasing concentrations of recombinant TRAIL (0-100 ng/mL) for 48 h, and cell viability was determined based on membrane integrity as previously described. Figure 1 shows that NCI-H460 cells were sensitive to TRAIL: their viability decreased in a dose-dependent manner. Markedly decreased cell viability was observed at ≥50 ng/mL TRAIL, and more than 30 % of the cell population was dead after 48 h of TRAIL treatment. However, A549 cells were resistant to TRAIL cytotoxicity: they maintained greater than 90 % viability even at 100 ng/mL TRAIL. This differential TRAIL cytotoxicity between the A549 and NCI-H460 human lung cancer cell lines was consistent with previous reports [25, 26].

### EEDS upregulated TRAIL receptors in A549 lung cancer cells

**Fig. 2:**
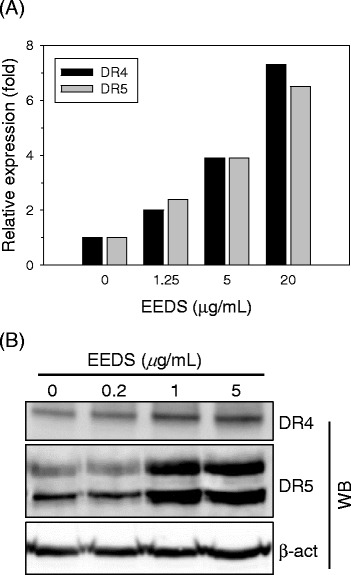
EEDS-mediated upregulation of TRAIL receptors. **a** A549 cells were treated with various concentrations of EEDS. After 24 h, total RNA was prepared from the cells, and changes in relative gene expression in response to EEDS were determined by expression chip analysis as described in our previous report [24]. The relative expression of two human TRAIL receptors (DR4, black bars; DR5, grey bars) in the treatment group is presented compared to vehicle treatment (PBS containing 0.1 % DMSO). **b** A549 cells were exposed to increasing concentrations of EEDS (0-5 μg/mL). After 24 h, DR4 and DR5 protein expression was determined by western blot analysis. β-actin was included as a loading control

We previously determined the dose-dependent effects of EEDS on global gene expression in A549 cells using microarray analysis [24]. From this gene expression analysis, we identified two human TRAIL receptors, DR4 and DR5, that were upregulated in a dose-dependent manner by EEDS. The increased DR4 and DR5 expression was observed at the low concentration of 1.25 μg/mL EEDS, and DR4 and DR5 expression increased nearly 7-fold in response to 20 μg/mL EEDS after 24 h treatment when compared to vehicle treatment (0.1 % DMSO; Fig. 2a). To confirm the EEDS-mediated upregulation of DR4 and DR5, we utilized western blot analysis to determine the intracellular change in DR4 and DR5 protein expression in response to EEDS. As shown in Fig. 2b, DR4 and DR5 protein expression increased in a dose-dependent manner in response to EEDS, and the intracellular protein levels of DR4 and DR5 began to increase at 1 μg/mL EEDS.

### EEDS sensitized A549 lung cancer cells to TRAIL

**Fig. 3:**
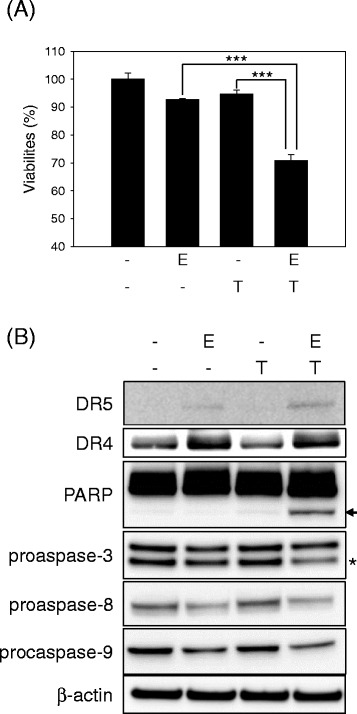
EEDS enhanced TRAIL toxicity in A549 lung cancer cells. **a** TRAIL-refractory A549 cells were treated with a combination of EEDS (E, 5 μg/mL) and TRAIL (T, 100 ng/mL) for 24 h. Cell viability was determined based on membrane integrity as previously described. The data are presented as the mean ± SD of triplicate experiments. ***P <0.001. **b** Enhanced apoptotic cell death in A549 cells by co-treatment of EEDS (E, 5 μg/mL) and TRAIL (T, 100 ng/mL) for 24 h. Whole cell lysates were prepared and subjected to western blot analysis using specific antibodies. The arrow indicates the cleaved forms of PARP. Asterisk indicates the specifically bound proteins of procaspase-3. β-actin was included as a loading control

After observing the increased expression of the TRAIL death receptors, DR4 and DR5, in response to EEDS, we determined whether EEDS increased TRAIL toxicity in A549 lung cancer cells. A549 cells treated with EEDS (5 μg/mL) or TRAIL (100 ng/mL) alone maintained greater than 90 % viability after 24 h. However, cell viability decreased significantly 70 % when A549 cells were co-treated with EEDS and TRAIL (Fig. 3a). We next determined whether apoptotic cell death was enhanced by co-treatment with EEDS and TRAIL. A549 cells were treated with a combination of EEDS (5 μg/mL) and TRAIL (100 ng/mL) for 24 h, and the protein expression of apoptosis-related intracellular proteins was determined by western blotting. As shown in Fig. 3b, the expression of DR4 and DR5 by was induced in A549 cells by EEDS as expected. Treatment of cells with EEDS or TRAIL alone for 24 h did not induce the cleavage of PARP protein in TRAIL-treated cells. In contrast, co-treatment of cells with EEDS and TRAIL led to a marked induction of apoptotic cell death and activation of caspase-3/8/9, which were revealed by cleavage of intact PARP and procaspases, respectively. Taken together, these results indicated that EEDS sensitized A549 lung cancer cells to TRAIL-induced apoptotic cell death by upregulating TRAIL receptors.

### Upregulation of TRAIL receptors by EEDS was mediated by CHOP

**Fig. 4:**
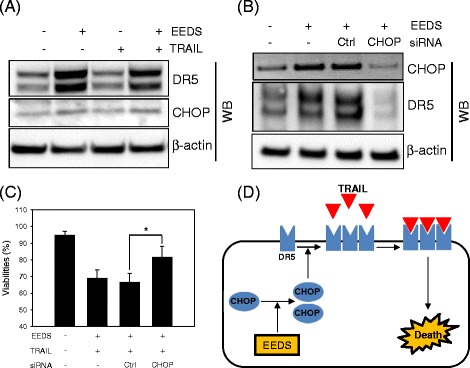
CHOP mediated the DR5 upregulation by EEDS. **a** A549 cells were treated with a combination of EEDS (E, 5 μg/mL) and TRAIL (T, 100 ng/mL) for 24 h, and the intracellular expression levels of DR5 and CHOP were determined by western blot analysis. **b** A549 cells were transiently transfected with control or CHOP siRNA. After 48 h, the cells were treated with 5 μg/mL EEDS for 24 h. Whole cell lysates were prepared, and the intracellular expression levels of DR5 and CHOP were determined by western blot analysis. β-actin was included as a loading control. **c** CHOP knockdown inhibited EEDS-enhanced TRAIL toxicity. A549 cells were transfected with control or CHOP siRNA for 48 h and then treated with a combination of EEDS (5 μg/mL) and TRAIL (100 ng/mL). Cell viability was determined after 24 h of drug treatment as previously described. The data are presented as the mean ± SD of triplicate experiments. *P <0.05. **d** Schematic of the EEDS-mediated sensitization of A549 lung cancer cells to TRAIL

Certain previous studies have revealed that the drug-induced sensitization of cancer cells to TRAIL occurs through the upregulation of TRAIL receptors, especially DR5, which is mediated by the ER stress-induced transcription factor CHOP [19, 28, 29]. Therefore, we investigated whether EEDS affected CHOP expression in A549 cells. A549 cells were treated with a combination of EEDS (5 μg/mL) and TRAIL (100 ng/mL) for 48 h, and changes in CHOP and DR5 protein expression were determined by western blot analysis. CHOP protein expression increased in response to EEDS (Fig. 4a). However, TRAIL did not affect CHOP expression alone or in a combination with EEDS. A dose-dependent increase in intracellular CHOP mRNA expression was observed in our previous gene chip analysis of EEDS-treated A549 cells [24]. Next, we utilized CHOP siRNA to determine whether CHOP knockdown inhibited EEDS-induced DR5 upregulation. Commercially available siRNA against CHOP successfully decreased intracellular CHOP protein expression (Fig. 4b). DR5 expression was also downregulated by CHOP siRNA, demonstrating that CHOP is a key mediator of EEDS-induced DR5 upregulation. The engagement of CHOP in EEDS-induced DR5 upregulation and apoptotic cell death was further confirmed by the recovery of cell viability from the synergistic cytotoxicity of EEDS and TRAIL by pre-treatment of CHOP siRNA (Fig. 4c). Figure 4d summarizes our findings that EEDS treatment increased the expression of transcription factor CHOP, which in turn induced the expression of TRAIL death receptors, such as DR5; more recombinant TRAIL molecules bound to the increased number TRAIL receptors, ultimately enhancing apoptotic cell death.

## Discussion

TRAIL is as a promising anticancer therapy that lacks serious side effects due to its ability to selectively kill cancer cells without harming most normal cells. However, some highly malignant tumors are resistant to TRAIL-induced programmed cell death [30]. The mechanisms underlying TRAIL resistance include decreased expression or dysfunction of DR4 and DR5, defects in the DISC that comprises FADD and caspase-8, overexpression of Bcl-2, Bcl-X_L_, FADD-like interleukin-1β-converting enzyme-inhibitory protein or inhibitor of apoptosis protein, dysfunction of Bax and Bak, decreased release of second mitochondria-derived activator of caspases (Smac/Diablo) into the cytosol, and constitutive activation of survival signaling molecules, such as mitogen-activated protein kinase, Akt, and nuclear factor-kappa-B [20, 30]. Therefore, we hypothesized that TRAIL resistance in cancer cells can be overcome by reversing the mechanisms by which TRAIL resistance is established, such as by upregulating DR4 and DR5, overexpressing pro-apoptotic proteins, downregulating anti-apoptotic proteins, and inhibiting key factors that regulate cell survival.

As a complex disease, cancer etiology involves multiple risk factors; therefore, the treatment of cancer may require a multi-target approach. Complementary and alternative medicine, which has traditionally used crude extracts or fractions of medicinal herbs, may offer new opportunities for cancer treatments involving a multi-component and multi-target strategy [3]. In this study, we were the first to demonstrate that the crude extract of *D. sophia* seeds effectively sensitized TRAIL-refractory A549 lung cancer cells to TRAIL-induced apoptosis by upregulating DR5 at the transcriptional level (Figs. 2 and 3). *D. sophia* (L.) Webb ex Prantl (Flixweed) is an annual weed that belongs to the Brassicaceae (Cruciferae) family. It is widely distributed throughout Europe and temperate to tropical Asian countries and produces large numbers of tiny red to brown seeds (0.7–1.5 mm long) from early to late summer [31]. In traditional folk medicine, different parts of *D. sophia* have been used to treat jaundice, febrifuge, laxative, and furuncle in Middle Asia [32] and for cough, edema, asthma, heart disease, and cancer in China [33, 34]. A variety of phytochemicals have been identified from the extracts of aerial parts and seeds of *D. sophia*. They are small molecules (amino acids, alcohols, aldehydes, ketones), cardiac glycosides (erysimoside, evobioside, helveticoside, strophanthidin), coumarins (bergapten, isoscopoletin, psoralene, scopoletin, xanthotoxin, xanthotoxol), fatty acids (arachic acid, capric acid, eicosenoic acid, erucic acid, lauric acid, linoleic acid, linolenic acid, myristic acid, oleic acid, palmitic acid, stearic acid), flavonoids (drabanemoroside, isoquercitrin, isorhamnetin, isorhamnetin–3-O-β-D-glucopyranoside, kaempferol, quercetin, quercetin 3-O-α-L-rhamnopyranosyl-(1 → 2)-α-L-arabinopyranose, quercetin-3-O-β-D-glucopyranoside), flavonol glycoside (artabotryside A), glucosinolates (gluconapin, sinigrin), lactones (descurainolide A and B), lignan (syringaresinol), nor-lignan (descuraic acid), lipids (epoxyacylglyceride, triacylglyceride), phenolic compounds (3,4,5-tritrimethoxy cinnamic acid, isovanillic acid, *p*-benzoic acid, *p*-hydroxybenzaldehyde, sinapic acid, syringic acid), phytosterol (daucosterol), sinapoyl glycosides (1,2-di-O-sinapoyl-β-D-glucopyranose, 1,2-disinapoylgentiobiose, 1,3-di-O-sinapoyl- β-D-glucopyranose), and a unique group of compounds (descurainin, descurainin A, descurainoside, descurainoside A and B) [34–40]. The biological activities of the extract of *D. sophia*, such as analgesic, antipyretic, anti-inflammatory, and cytotoxic effects may be attributed to some of aforementioned phytochemicals or unknown ones, and/or their combination. In the present study, however, we did not identify the active component(s) of EEDS responsible for the synergistic anticancer effect with TRAIL; this discovery remains for future study. The active components of EEDS can be identified using conventional activity-guided fractionation of crude extracts and spectroscopic analyses of isolated single phytochemicals.

We also demonstrated that the EEDS-induced upregulation of DR5 was mediated by CHOP, as revealed by the efficient inhibition of EEDS-induced DR5 expression by CHOP siRNA. CHOP is a transcription factor that is induced by various stresses, including ER stress (the unfolded protein response) [29] and reactive oxygen species (ROS) [41]. Since the relationship between CHOP and DR5 was discovered in 2004 [42], numerous studies have shown that the induction of CHOP by stressful stimuli mediates DR5 upregulation during TRAIL sensitization via a consensus CHOP-binding element (GAGGATTGCGATC) in the DR5 promoter [42, 43]. Biologically active phytochemicals isolated from medicinal plants or their crude extracts have been reported to enhance TRAIL-induced apoptosis via CHOP-dependent DR5 upregulation in cancer cells [21, 29]. In addition to the CHOP-dependent pathway, CHOP-independent DR5 upregulation has been observed in certain cancer cells treated with TRAIL and other chemicals, such as Orlistat (Xenical^TM^) [44] and curcumin [45], although the key mediators of DR5 upregulation have not been identified.

## Conclusions

In summary, our findings suggested a future model of integrative anticancer therapy that incorporates a more stable recombinant TRAIL or agonistic ligand targeting TRAIL receptors with traditional herbal medicine to sensitize refractory tumors to TRAIL toxicity. In this aspect, EEDS is a suitable initial material for the development of an herbal supplement for anticancer therapy. Further studies should be performed to validate the antitumor efficacy of the combination therapy of TRAIL and EEDS using a suitable in vivo animal model and to identify the active component of EEDS that is responsible for the TRAIL synergism.

### Availability of data and materials

All datasets supporting our findings are available and presented as an Additional file 1.

## Additional file

Additional file 1: The original dataset supporting the findings in this study. (PPTX 477 kb)

## Abbreviations

Bid: BH3-interacting domain death agonist
BSA: bovine serum albumin
c-FLIP: cellular FADD-like interleukin-1β-converting enzyme inhibitory protein
CHOP: CCAAT/enhancer-binding protein homologous protein
DISC: death-inducing signaling complex
DMSO: dimethyl sulfoxide
DR4: death receptor 4
DR5: death receptor 5
EEDS: ethanolic extract of D. sohia seeds
ER: endoplasmic reticulum
FADD: Fas-associated death domain protein
NSCLC: non-small cell lung carcinoma
PARP: poly ADP ribose polymerase
PBS: phosphate-buffered saline
siRNA: small interfering RNA
SD: standard deviation
TNF: tumor necrosis factor
TRAIL: tumor necrosis factor-related apoptosis-inducing ligand
