# Supplementary material for: Ethanolic extract of Descurainia sophia seeds sensitizes A549 human lung cancer cells to TRAIL cytotoxicity by upregulating death receptors
Source: BMC Complement Altern Med. 2016 Apr 2;16:115. doi: 10.1186/s12906-016-1094-0 (PMC4818868; doi:10.1186/s12906-016-1094-0)
Supplement: Additional file 1: — The original dataset supporting the findings in this study. (PPTX 477 kb) [file 12906_2016_1094_MOESM1_ESM.pptx]

## Slide 1
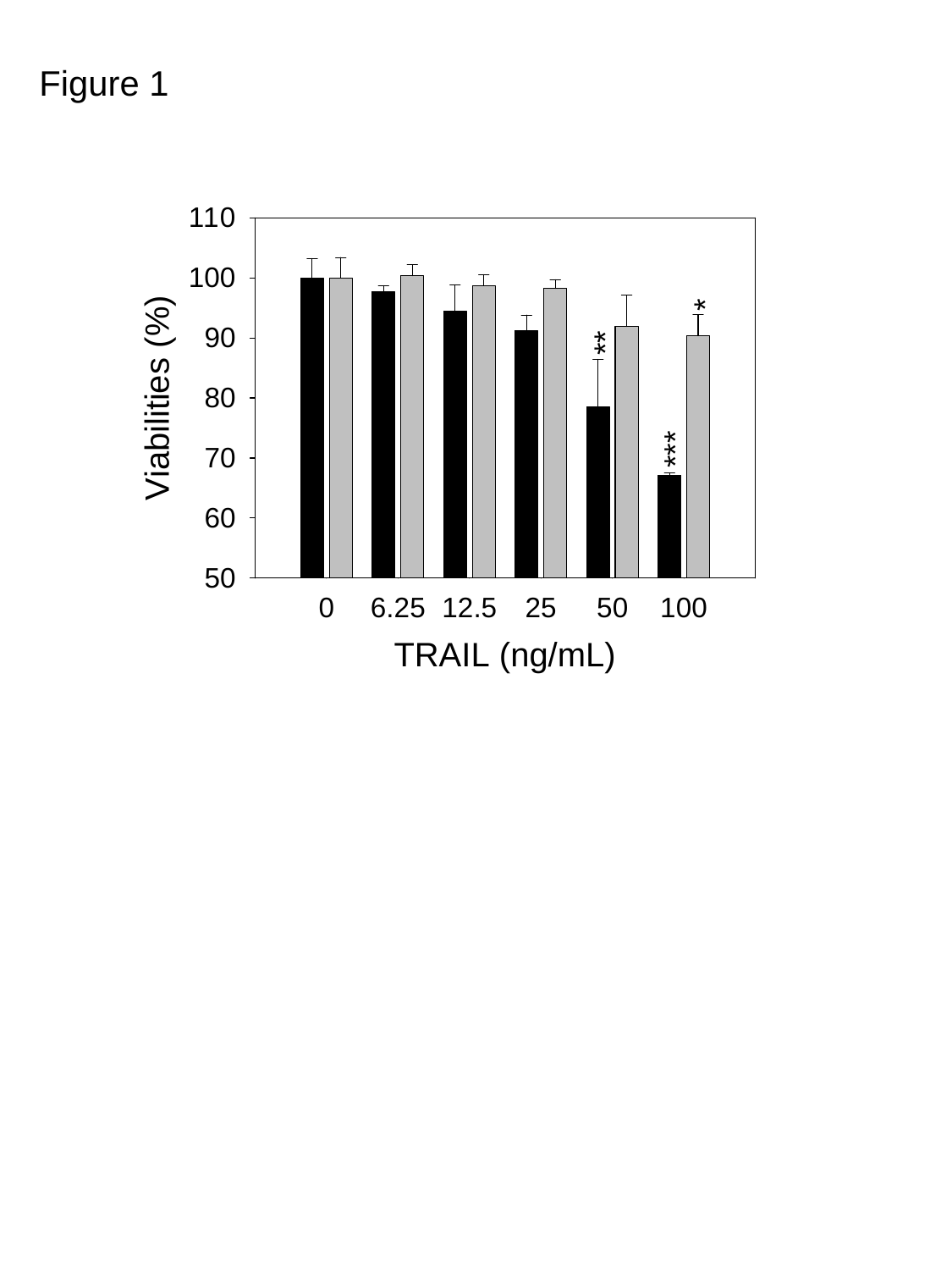

Figure 1
*
**
***

## Slide 2
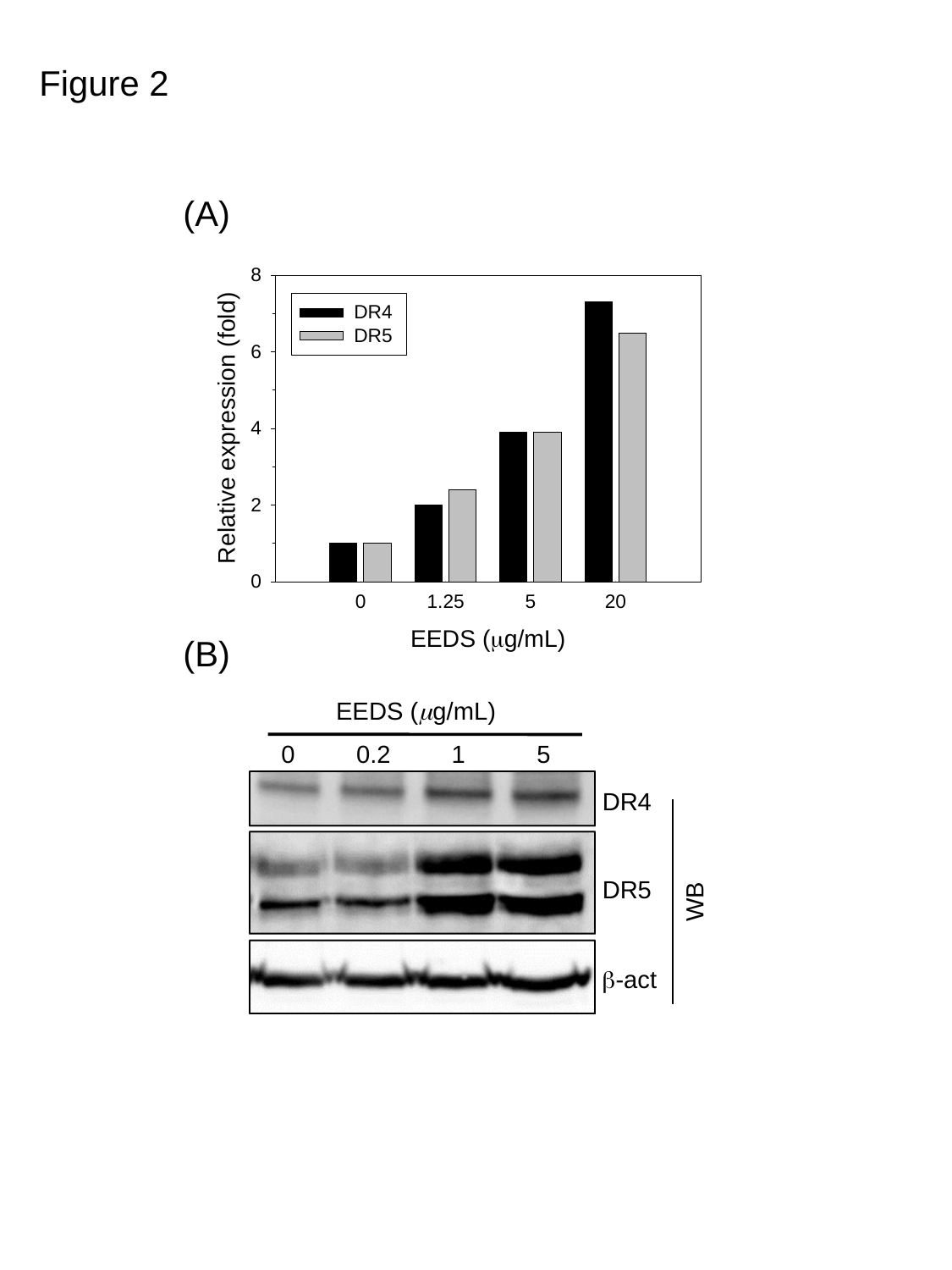

Figure 2
(A)
(B)
| EEDS (mg/mL) | | | |
| --- | --- | --- | --- |
| 0 | 0.2 | 1 | 5 |
DR4
DR5
WB
b-act

## Slide 3
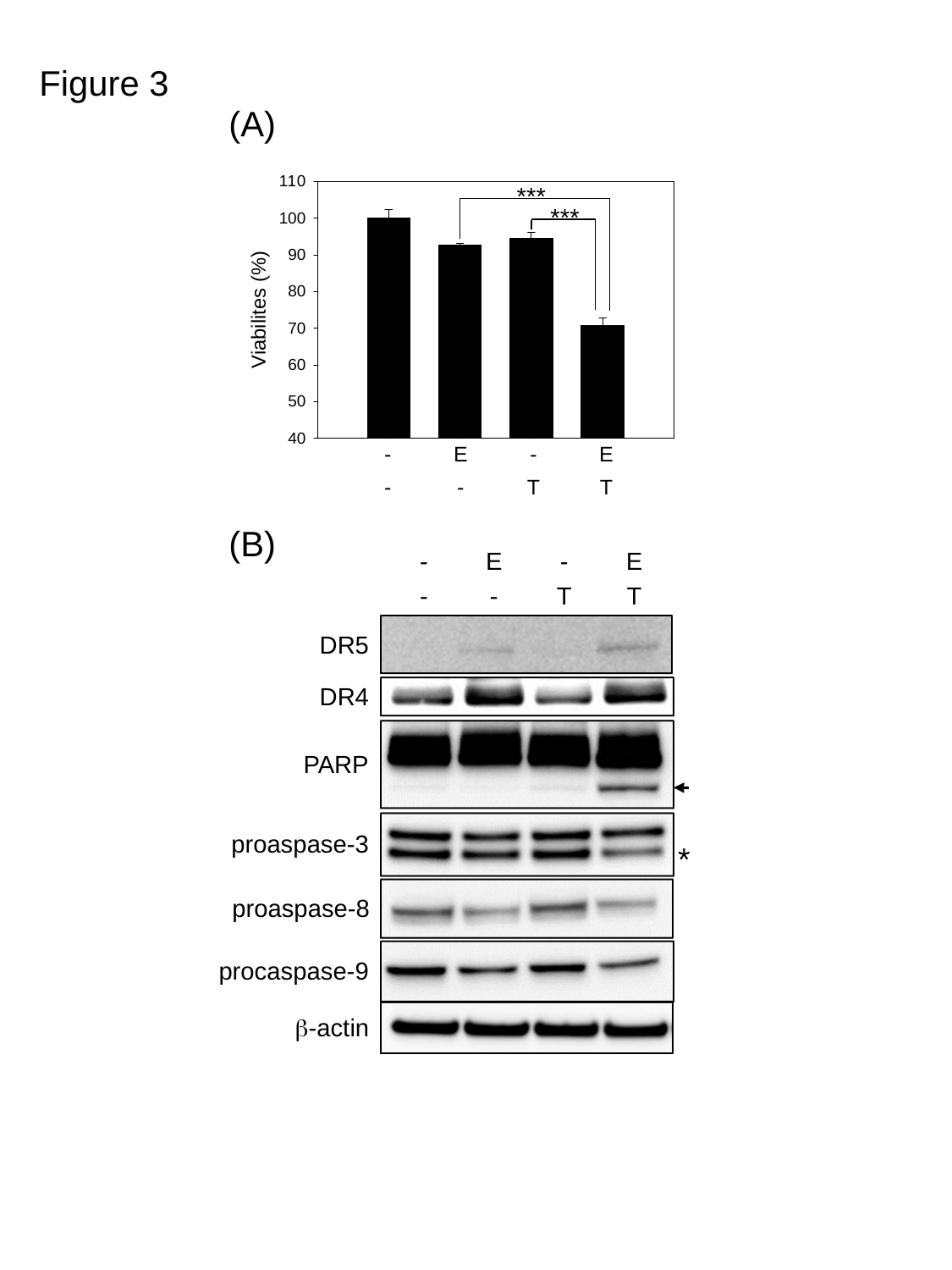

Figure 3
(A)
***
***
| - | E | - | E |
| --- | --- | --- | --- |
| - | - | T | T |
(B)
| - | E | - | E |
| --- | --- | --- | --- |
| - | - | T | T |
DR5
DR4
PARP
proaspase-3
*
proaspase-8
procaspase-9
b-actin

## Slide 4
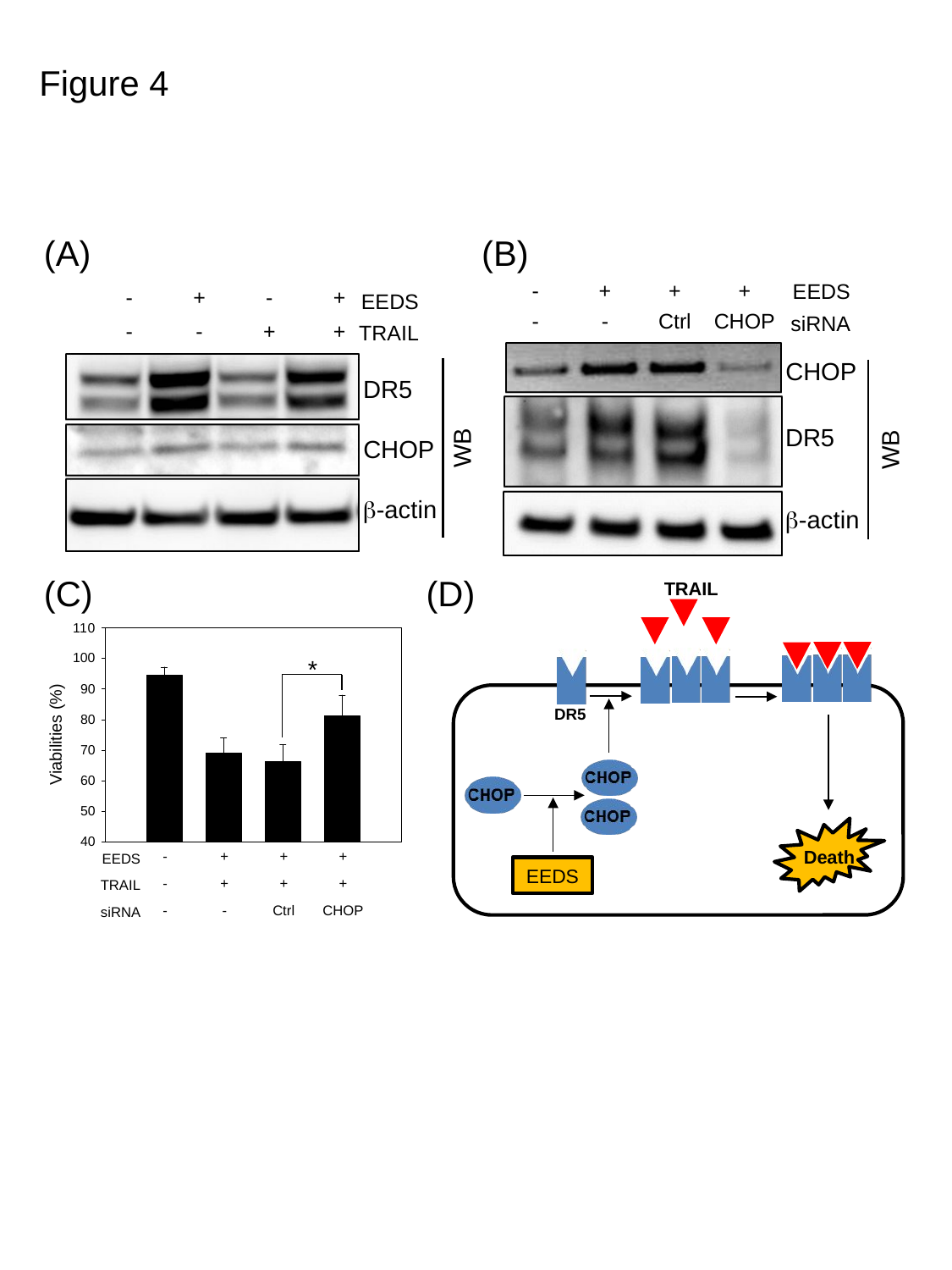

Figure 4
(A)
(B)
| | | | |
| --- | --- | --- | --- |
| - | + | - | + |
| - | - | + | + |
EEDS
| - | + | + | + |
| --- | --- | --- | --- |
| - | - | Ctrl | CHOP |
EEDS
siRNA
TRAIL
CHOP
DR5
DR5
WB
CHOP
WB
b-actin
b-actin
(C)
(D)
TRAIL
*
DR5
Death
EEDS
| - | + | + | + |
| --- | --- | --- | --- |
| - | + | + | + |
| - | - | Ctrl | CHOP |
EEDS
TRAIL
siRNA
